# Shaping Electromagnetic Waves with Flexible and Continuous Control of the Beam Directions Using Holography and Convolution Theorem

**DOI:** 10.1038/s41598-019-48301-2

**Published:** 2019-08-14

**Authors:** Majid Karimipour, Nader Komjani, Iman Aryanian

**Affiliations:** 1 Iran University of Science and Technology (IUST) Department of Electrical Engineering, Tehran, +98 Iran; 2 Iran Telecommunication Research Center, Tehran, 1439955471 Iran

**Keywords:** Electrical and electronic engineering, Electronic and spintronic devices

## Abstract

In this article, several versatile electromagnetic (EM) waves are presented with predefined shapes and directions based on the holography and convolution theorem. Inspiring the holography theory, a reflective interferogram is characterized by interfering the near field distributions of the object and reference waves. In this regard, the interference pattern on the hologram could be viewed as the inverse Fourier transform of the object and reference waves. Therefore, the capability of steering the EM shaped beam is realized using the convolution theorem (as an interesting property of the Fourier transform), which makes a link between the hologram impedance-pattern and far-field pattern domains. The main advantage of incorporating the holography concept and convolution theorem is realizing arbitrary shaped-beam EM waves with the possibility of flexible manipulation of the beam directions without employing any optimization algorithm and mathematical computation. It is demonstrated that the method could implement a combination of simple beams (such as collimated beams) and complex beams (such as cosecant squared, flat top, isoflux beams, etc.) with each beam possessing arbitrary direction by the same design topology. To experimentally verify the concept, a prototype of the hologram with three separate beams including two tilted cosecant squared shaped beam and one broadside pencil beam is fabricated and measured. The measured results show a significant agreement between theoretical findings.

## Introduction

In modern technology, design of devices with the capability of shaping the electromagnetic (EM) waves is rapidly progressing. Understandably, as far as the far field is concerned, metasurface structures are good candidates for manipulating the EM features of the waves, due to having desirable capabilities for engineering the amplitude and phase of the waves^1–3^. Therefore, it is more convenient for metasurfaces to create versatile radiation beams. In general, metasurfaces, which are the planar version of bulky 3D metamaterials, are divided into two categories including periodic and quasi-periodic structures. The first category is widely used in radar cross section reduction^4^ by developing perfect absorbers^5^, polarization rotators^6–8^, frequency selective structures^9^, polarization converters^10^ and artificial magnetic conductors. Meanwhile, the quasi-periodic metasurface has become more attractive for scientists, by virtue of providing more design freedom to perform inhomogeneous surfaces. More recently, many novel EM functions like beam steering^11^, beam shaping^12^ and focusing^13–15^, anomalous reflection and refraction governed by the generalized Snell’s law^16^, space wave to surface wave converters and vice versa have been introduced^17,18^. The metasurface in reflection and transmission mode, which is the case in this article, is one of the best solutions to fully control space waves from the aspect of wave front shape^19^, spin^20^ and orbital angular momentums^21^, etc. Many rapidly evolving devices like reflectarray^22–26^, and transmitarray^27^, which their design equations being formulated by the generalized Snell’s law, are some of the most important planar metasurfaces working in transmission and reflection modes. For metasurfaces with reflectarray operation, the surface should be impenetrable and the reflection phase can be characterized by the impedance surface dyadic, which is generated by a dense grid of metallic particles over a grounded dielectric surface^28^.

Up to the authors’ knowledge, the most reliable method of shaping EM waves is based on optimization techniques including local and global search algorithms with iterative nature. This is done by locally adjusting the phase of reflection and transmission waves in a quasi-periodic metasurface-based structure. This common method is known as the phase only synthesis method^29,30^. Apparently, in this mode, both induced electric and magnetic averaged surface currents should simultaneously exist to fully control the reflection phase of waves.

The emergence of the holography concept, which was demonstrated as a two-step imaging process, in the microwave domain, opened a new insight into wave phenomena, specifically for wave propagation leading to development of a new class of antenna named holographic antenna^31^. In most cases, the metasurface-based holographic radiators in the microwave regime are implemented practically with artificial impedance surfaces which are usually constructed with multiple wavelength long structures. To provide a more insightful interpretation of how holographic metasurface-base radiators work, it is necessary to explain the two-step imaging process, including the hologram formation and wave front reconstruction steps, in the microwave regime. In this fashion, some terminologies such as reference and object waves are now common. The reference wave illuminates the hologram and is coherent with the wave scattered from the object, which is called object wave. At the hologram formation step, a reference wave and an object wave interact with each other on a certain plane in such a way that the phase and amplitude of the field scattered from the object could be detected. At the reconstruction step, a reference wave coherent with that of the scattered wave from the object is needed. The bothersome shortcoming in the holography method is the practical implementation of the hologram, especially when the polarization of the waves becomes prominent leading to generation of dyadic impedance surfaces. Several efforts have been reported until now about using the holography concept in the microwave regime, including planar and compact metasurface-based designs and reflector-based holography designs^31–40^.

In this article, combining the holographic principle and convolution theorem, we present a reflector-based metasurface with the capability of generating multiple predefined shaped beams at any directions. Recently, the concept of the convolution operation and its functionality for directing EM waves have been described in coding metasurfaces, in which the beam direction is limited by the periodicity of a gradient coding sequence^20,41^. In reflector-based metasurfaces corresponding to the holography theory, the interferogram is generated based on the near field data relevant to object and reference waves, which are the desired far-field radiation patterns and illuminated wave from a certain source, respectively. Therefore, one can conveniently make a link between the hologram impedance-pattern and far-field pattern domains using the Fourier transform, and consequently control the beam direction with a famous theorem named convolution. Given that the hologram can be realized by a continuous impedance profile, the scattered field direction can be determined at will. Several shaped beams like flat top, isoflux, squared cosecant and a combination of shaped and pencil beams are demonstrated. A prototype of a multiple beam radiator with two tilted squared cosecant beams and one broadside beam is fabricated as a proof of concept.

## Results

### The holography concept concept and convolution theorem

**Figure 1:**
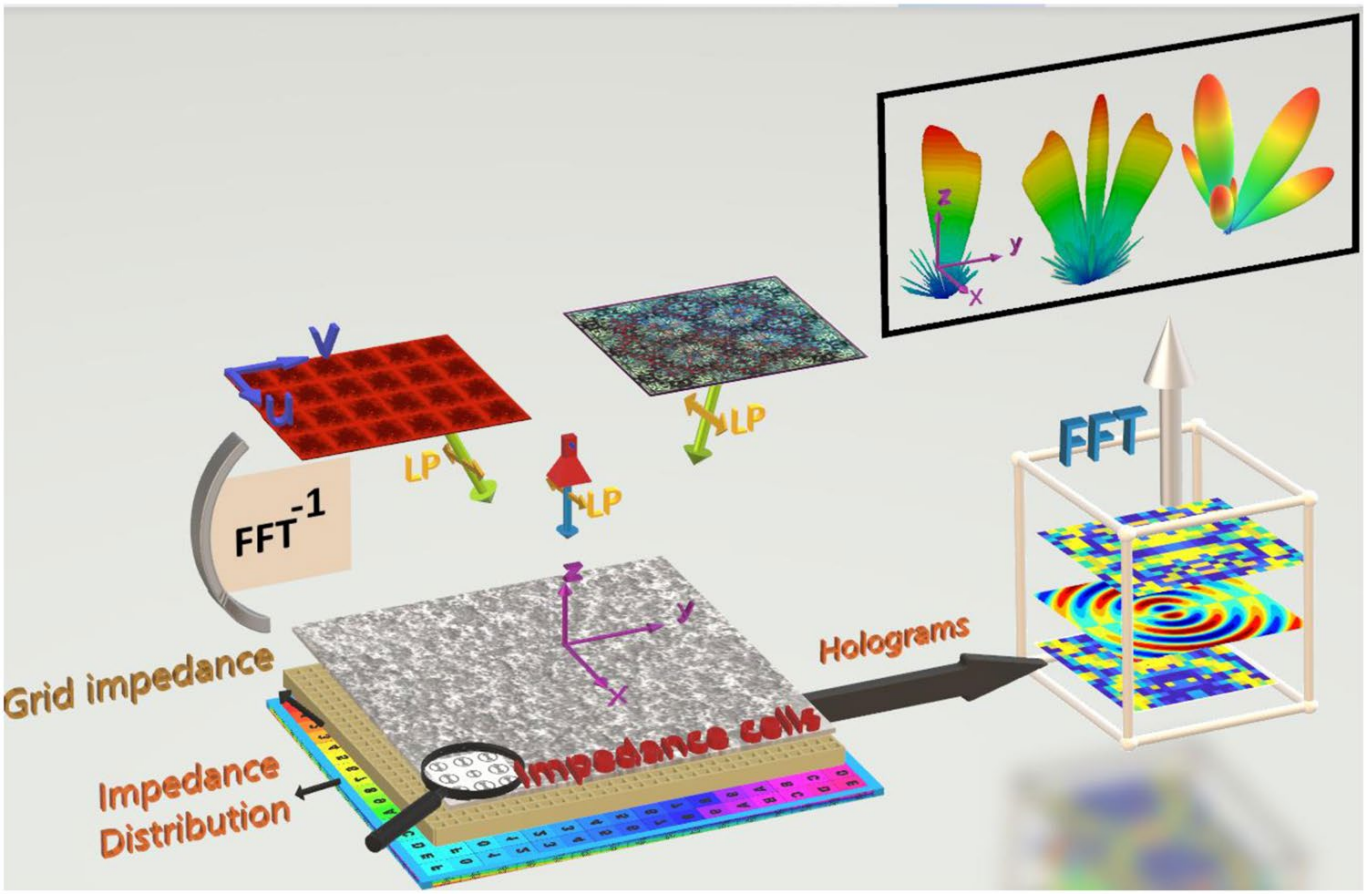
The conceptual demonstration of the holographic-based method for shaping the EM waves.

The conceptual scheme of the holographic-based method for shaping the EM waves is presented in Fig. 1. According to the theory of holography in the microwave regime, the hologram surface can be fully characterized with an impedance profile defined based on reference and object waves as

1 $$Z(x,y)=j[X+(M/n\,){\rm{Re}}(\mathop{\sum }\limits_{i=1}^{n}\,{\psi }_{rad}^{i}){\psi }_{ref}^{\ast }]$$

The quantities ${\psi }_{rad}^{i}$ and *ψ*_*ref*_ are the near field distributions of the *i*^*th*^ object and reference waves on the hologram surface, respectively^33^, and *n* is the number of object beams. By definition, the parameters *X* and *M* are the average impedance and modulation depth, respectively. Note that in reflector-based holograms, *Z*(*x*, *y*) accommodates with the input impedance concept, which is the ratio of tangential electric field to tangential magnetic field of the space wave near the reflecting surface. This is why, as opposed to conventional holographic structures, we treat a space wave instead of a surface wave in reflector-based holograms^39^. Specifically, as expressed in leaky wave structures, if object waves are of pencil beam type, the relevant near field distribution of the waves will be in form of *rect* function with a uniform profile, and hence, the hologram for multiple object beams is characterized with the superposition of the corresponding *rect* functions. This fact can further be confirmed by the Fourier transform operation. For general-shape patterns as object waves, one can plainly determine the functions ${\psi }_{rad}^{i}$ by applying an inverse Fourier transform operation and consequently benefiting from the convolution theorem as an interesting feature of the Fourier transform in the design process^42,44^. For more explanation, the following equivalence can be established between the far-field pattern domain defined in the (u, v) plane and the hologram pattern domain as follows

2 $$f({x}_{\lambda },{y}_{\lambda })g({x}_{\lambda },{y}_{\lambda })\mathop{\iff }\limits^{fft}F(u,v)\ast G(u,v)$$

In (2), *u* = sin*θ* cos*φ* and *v* = sin*θ* sin*φ* are defined in an angular coordinate system, the functions *f* and *g* are defined in the hologram coordinate system and the functions *F* and *G* are the spectral domain representation of the original functions *f* and *g*. In addition, *x*_*λ*_ = *x*/*λ* and *y*_*λ*_ = *y*/*λ*are the electrical lengths. Let the function *g* be a special function with uniform amplitude and gradient phase along the x and y directions. Therefore, by evoking the convolution theorem, the relation (2) could be rewritten as follows

3 $$f({x}_{\lambda },{y}_{\lambda }){e}^{j({x}_{\lambda }{u}_{0}+{y}_{\lambda }{v}_{0})}\mathop{\iff }\limits^{fft}F(u,v)\ast \delta (u-{u}_{0},v-{v}_{0})=F(u-{u}_{0},v-{v}_{0})$$

where *δ*(.) is the kronecker delta function and (*u*_0_, *v*_0_) = (sin*θ*_0_ cos*ϕ*_0_, sin*θ*_0_ sin*ϕ*_0_). Comparing the relations (3) and (1), we can deduce that if the illustrative function of the hologram impedance profile is multiplied by the well-behaved function ${e}^{j({x}_{\lambda }{u}_{0}+{y}_{\lambda }{v}_{0})}$, the spectral counterpart of the descriptive function will be tilted by (*u*_0_, *v*_0_) in the (u, v) plane. This interesting property allows manipulating the far-field radiated wave in arbitrary direction with continuous values. In general, the function *ψ*^*i*^ can be extracted from

4 $${\psi }^{i}(x,y)={\rm{ifftshift}}\langle {\rm{IFFT}}2[\frac{{f}_{0}(r){E}_{copol}^{far\,field}(u,v){f}_{1}(u,v)}{{f}_{2}(u,v)}]\rangle $$

In (4), ${E}_{copol}^{farfield}(u,v)$ is the co-pol component of the electric field defined in the (u, v) plane that can have horizontal or vertical polarization depending on the antenna design requirement. IFFT2 is the 2D inverse Fourier transform operator. Since in the pattern calculation the zero frequency point, existed in FFT routine, should represent the center point of the (u, v) plane, we have to select odd number of observation points in the (u, v) plane to satisfy this condition. In addition, Matlab’s 2D-IFFT command swaps the coordinates so that the zero frequency point is located at the lower left corner. After the 2D-IFFT command (IFFT2) is executed, it is necessary to correct the obtained results by using the ifftshift command, which corrects the quadrants so that the center of the (u, v) plane is placed in the center of the matrix^43^. Constituent functions of (4) are defined as follows^44^

5 $${f}_{0}(r)=\frac{2\pi r}{j{k}_{0}{e}^{-j{k}_{0}r}}$$

6 $${\rm{X}} \mbox{-} {\rm{Pol}}:\,{f}_{1}(u,v)=\frac{{u}^{2}+{v}^{2}}{[{u}^{2}+{v}^{2}\sqrt{1-{u}^{2}-{v}^{2}}]},\,{\rm{Y}} \mbox{-} {\rm{Pol}}:\,{f}_{1}(u,v)=\frac{{u}^{2}+{v}^{2}}{[{v}^{2}+{u}^{2}\sqrt{1-{u}^{2}-{v}^{2}}]}$$

The relation (6) determines the value of *f*_1_(*u*, *v*) depending on the polarization type of the desired object wave. Moreover, *f*_2_(*u*, *v*) = *N*_*x*_*N*_*y*_ϒ(*u*, *v*)ϒ′(*u*, *v*), where the quantities ϒ(*u*, *v*) and ϒ′(*u*, *v*) are read as

7 $$\Upsilon (u,v)={p}_{x}{p}_{y}\,\sin \,c(\frac{{k}_{0}u{p}_{x}}{2})\sin \,c(\frac{{k}_{0}v{p}_{y}}{2}),\,\Upsilon ^{\prime} (u,v)={e}^{-j\frac{{k}_{0}}{2}[u({N}_{x}-1){p}_{x}+v({N}_{y}-1){p}_{y}]}$$

In the above relations, the parameters *u*, *v*, and the mesh grid center points are defined as follows

8 $$u=\frac{2\pi }{{N}_{x}{p}_{x}{k}_{0}}p,\,v=\frac{2\pi }{{N}_{y}{p}_{y}{k}_{0}}q$$

9 $$p=-\,\frac{{N}_{x}}{2},-\,\frac{{N}_{x}}{2}+1,\,\ldots ,+\,\frac{{N}_{x}}{2}-1,\,q=-\,\frac{{N}_{y}}{2},-\,\frac{{N}_{y}}{2}+1,\,\ldots ,+\,\frac{{N}_{y}}{2}-1$$

10 $$\begin{array}{rcl}({x}_{m},{y}_{n}) & = & (-\frac{{N}_{x}{p}_{x}}{2}+(m+1/2{p}_{x}),-\frac{{N}_{y}{p}_{y}}{2}+(n+1/2{p}_{y})),\\ m & = & 0,1,\,\ldots ,{N}_{x}-1,\\ n & = & 0,1,2,\,\ldots ,{N}_{y}-1\end{array}$$

where *N*_*x*_ and *N*_*y*_ are the number of cells in the x and y directions on the hologram plane. Moreover, *p*_*x*_ and *p*_*y*_ are the period of cells in the x and y directions, respectively. It is worthwhile to underline that the number of unit cells in the (x, y) plane and observation points defined in the (u, v) plane should be equal. Therefore, as an effective remedy, one can extend the hologram grid and make a virtual mesh grid so that the number of observation points, which are usually considered 2^*n*^ with *n* = 7 or 8, is equal to mesh grid cells. In this state, the amplitude of *ψ*^*i*^ should be set to zero for the cells outside the hologram plane. This issue causes some errors; however, it is a necessary condition for implementing a Fourier transform operation with high resolution in the far-field domain.

### Implementation of the shaped-beam radiation pattern

**Figure 2:**
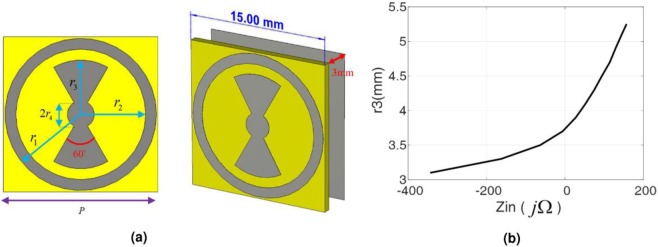
(**a**,**b**) Demonstration of the impedance unit cell geometry and corresponding geometrical parameters as *r*_1_ = 1.4*r*_3_, *r*_2_ = 1.2*r*_3_, *r*_4_ = 0.25*r*_3_, *r*_3_ = 2.5 *mm* : 0.2 *mm* : 5.25 *mm*. (**c**) The input impedance value as the parameter *r*_3_ changes.

Here, we aim to realize diverse radiation patterns based on the aforementioned holographic method. In all cases, the design frequency is considered 10 GHz and the hologram dimensions are 30 cm × 3 cm. The parameters *p*_*x*_ and *p*_*y*_ are selected to be 15 mm, which are equal to *λ*_0_/2 where *λ*_0_ is the free space wavelength. As a result, the hologram is divided into 400 equispace unit cells. A standard X-band pyramidal horn antenna with vertical polarization is applied as a point source emitting the reference waves. As a result, the quantity *ψ*_*ref*_ can be simply described as ${{\rm{\Psi }}}_{ref}=B{e}^{-j{k}_{0}r}$, where *B* is the amplitude of the spherical wave in the hologram plane, *k*_0_ is the free space wave number, and *r* = [(*x* − *x*_*f*_)^2^ + (*y* − *y*_*f*_)^2^ + (*H*)^2^]^0.5^ is the radial distance between the phase center of the horn and every point on the hologram, where (*x*_*f*_, *y*_*f*_, *H*) is the phase center coordinate of the horn. We placed the feed horn along the z-axis, *i.e*. (*x*_*f*_, *y*_*f*_) = (0, 0). The parameter *H* should be determined according to the efficiency considerations dictated by the focal-to-diameter ratio (*f*/*D*) parameter^43^. As such, for a horn with *q* = 10, *H* is equal to be 30.9 cm. It is worthwhile to point out that one can use the full wave simulation results of the near field components of the radiated electric fields of the horn on the hologram plane which leads to obtaining more precise data for *ψ*_*ref*_. The number of observation points in the spectral domain is selected 2^8^ × 2^8^. In order to realize the impedance profile characterized by (1), we used a combination of bowtie and circular ring metallic patches placed on a single layer of standard RT5870 high frequency laminate with the dielectric constant and thickness of 2.33 and 0.79 mm, respectively. The periodicity of the unit cell is selected to be 15 mm, which is equal to the mesh periodicity of the hologram defined above. The substrate layer is separated with a gap distance equal to 3 mm from the ground plane. Figure 2 shows the unit cell and simulated input impedance obtained in CST software by assuming periodic boundary condition. The design parameters are chosen so that no resonance point occurs in the impedance behavior of the element and consequently the parameter *X* is correctly valued. It is observed from Fig. 2 that the input impedance varies from −340 Ω to 175 Ω when *r*_3_ varies from 3.1 mm to 5.5 mm. Therefore, by selecting X = −82 Ω, and M = 257 Ω, the hologram surface is properly modulated. It is worth mentioning that when the desired far-field pattern is specified in each design, the quantity ${E}_{copol}^{farfield}(u,v)$ is determined. By introducing ${E}_{copol}^{farfield}(u,v)$ into (4), ${\psi }_{rad}^{i}$ will be obtained. On the other hand, *ψ*_*ref*_ is extracted from near field data of the horn in the center point of each cell on the hologram plane. Therefore, the amplitude and phase of the overall tangential electric field in the hologram plane, *i.e*, |Ψ_*ref*_. Ψ_*ref*_| and $\measuredangle ({{\rm{\Psi }}}_{ref}.{{\rm{\Psi }}}_{ref})$, and finally the impedance distribution of the hologram will be extracted from (1). This routine should be done accurately for each following example to implement the metasurface.

#### Flat top beam

**Figure 3:**
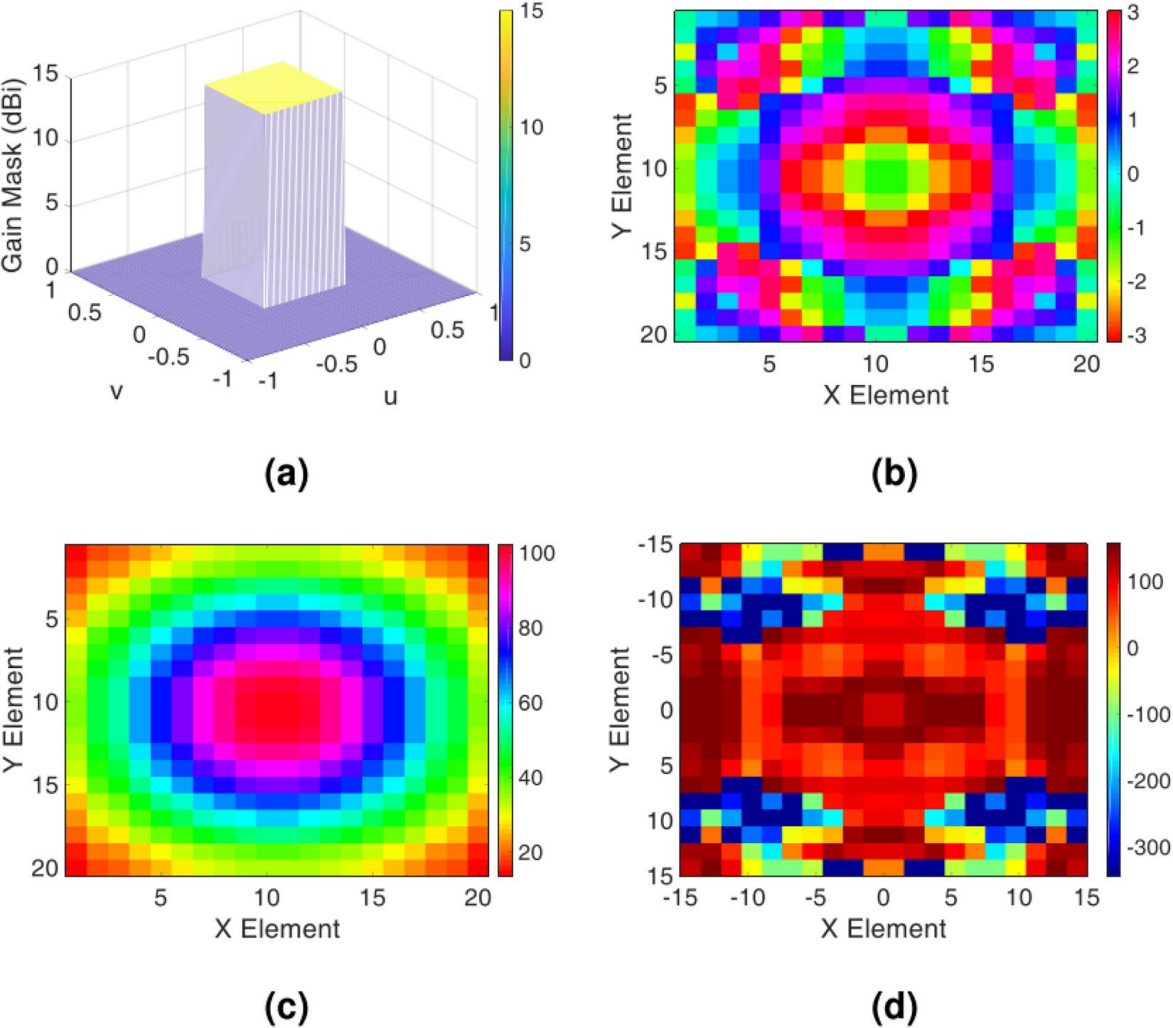
(**a**) The expected object wave in the (u, v) plane; (**b**) the phase and (**c**) amplitude of the overall tangential electric field in hologram plane; (**d**) the impedance distribution of the hologram obtained from the Eq. (1).

Initially, we perform a simple flat top pattern with the corresponding gain profile described in the spectral domain as ${G}_{copol}^{farfield(Y)}=15[{\bf{rect}}(u/{u}_{0},v/{v}_{0})]$, where *u*_0_ = *v*_0_ = 0.7. Figure 3a shows the desired object wave in the (u, v) plane. Following the extraction process of the impedance distribution outlined in the previous part, the requirements for the hologram implementation are provided. Figure 3b up to 3d present the overall tangential electric field distribution along with impedance distribution of the hologram. By mapping the obtained impedance distribution of the hologram onto the input impedance characteristic of the element shown in Fig. 2b, the element arrangement of the hologram surface is obtained.

**Figure 4:**
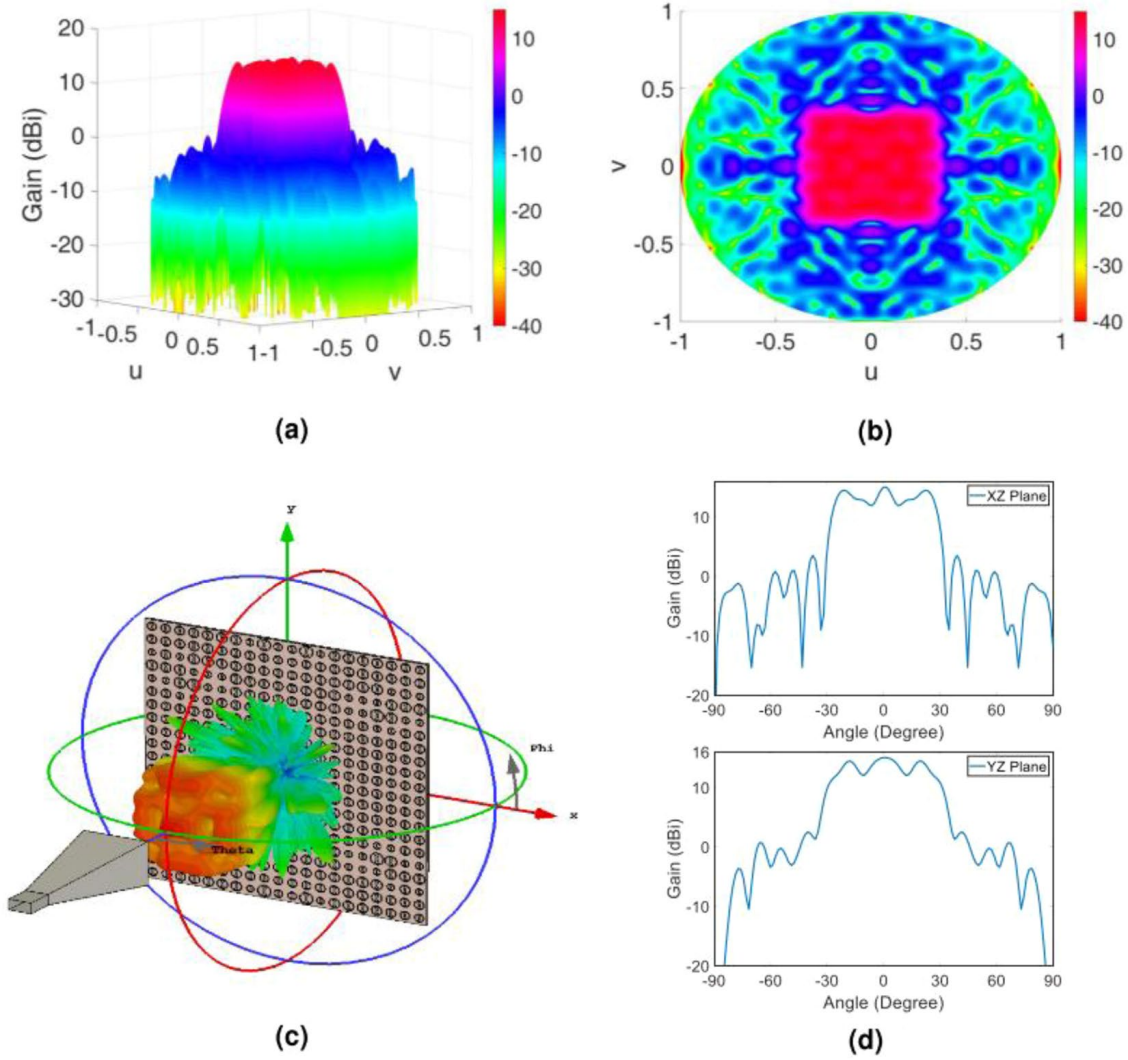
(**a**) Three-dimensional and (**b**) two-dimensional display of theoretical results of the object wave in the far field; (**c**) three-dimensional demonstration of the full wave simulation result of the object wave in CST software; (**d**) demonstration of the object wave in the two principal planes of the hologram. Note that the principal planes in d are the XZ- and YZ-planes or u = 0 and v = 0, respectively.

In order to predict the object wave profile when the hologram is illuminated by the reference wave at the reconstruction step as well as to gain more insight from the radiation performance of the hologram, we analytically extract the object wave. This is done by applying the FFT routine to the overall tangential electric field of the hologram. Figure 4a,b demonstrate the resulted object wave. In order to numerically evaluate the capability of the hologram to generate the predefined object wave, we employ the CST software and calculate the far-field wave when the hologram is fed by the X-band standard pyramidal horn with 15 dBi gain. The full wave simulation of the far-field pattern along with the gain profile in two principal planes of the hologram (u = 0 and v = 0) are shown in Fig. 4c,d. As can be observed, very good results are achieved and the object wave is rebuilt with a high accuracy. The reason behind the non-symmetric object wave in the simulation results is that the horn antenna provides a non-symmetric pattern.

#### Tilted squared cosecant

**Figure 5:**
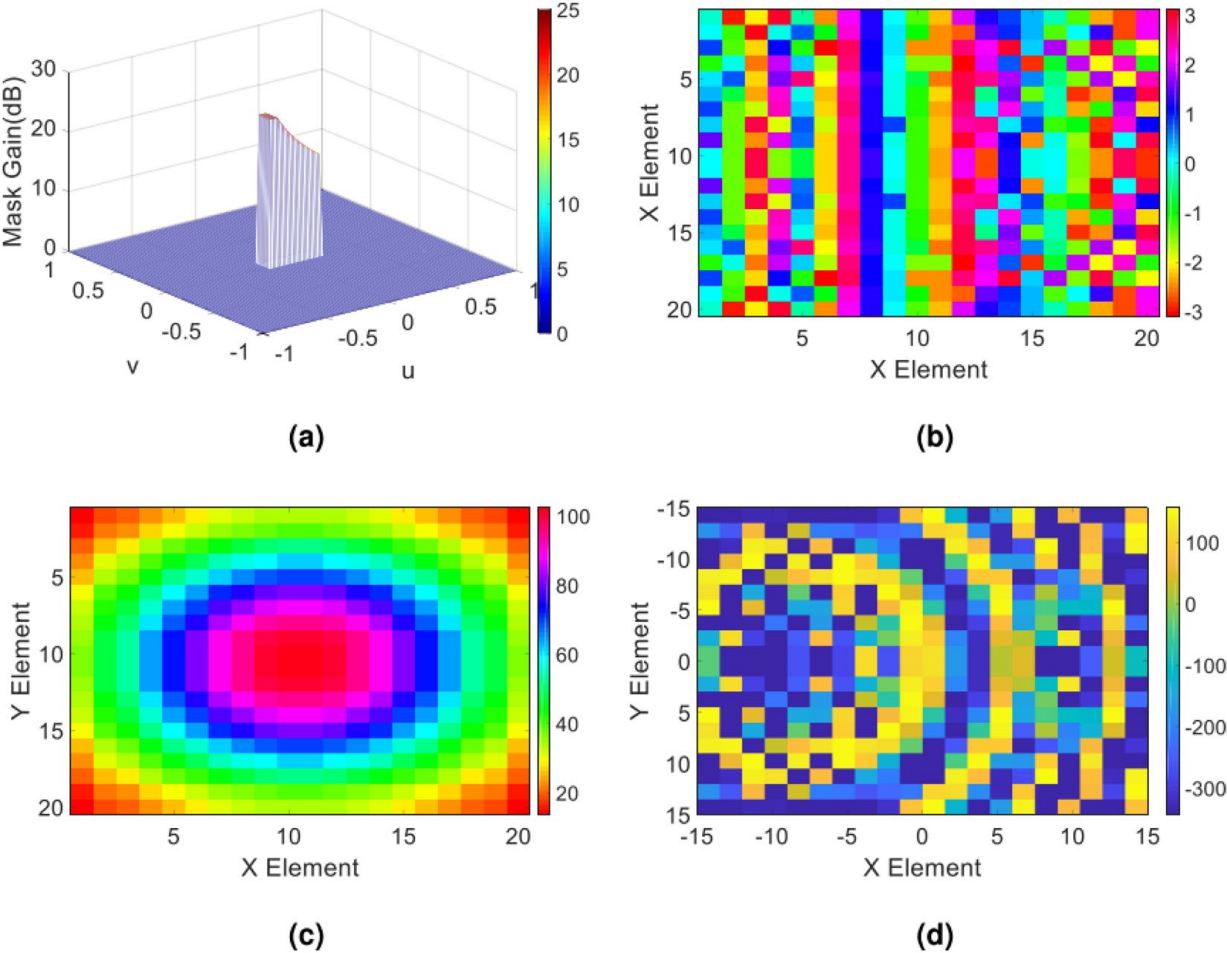
(**a**) The expected object wave in the (u, v) plane; (**b**) the phase and (**c**) amplitude of overall tangential electric field in the hologram plane; (**d**) the impedance distribution of the hologram obtained from the Eq. (1).

In this part, we aim to present an example which better clarifies the advantage of using the convolution theorem. In doing so, we expect that the metasurface radiates a tilted squared cosecant in the far-field region. Being inspired by the convolution theorem, we can define a non-tilted object wave in the form of squared cosecant and then multiply the impedance profile of the hologram with a proper function, as described in (3). Therefore, we can define

11 $${G}_{copol}^{far\,field(Y)}=[{\bf{rect}}(u/{u}_{0},v/{v}_{0})]\times [10\,\mathrm{log}(|\csc (\arcsin (u^{\prime} )){|}^{2}\,/{\rm{\max }}(|\csc (\arcsin (u^{\prime} )){|}^{2}))+25]$$

12 $$u^{\prime} =u\times {\bf{rect}}\frac{u-{u}_{1}}{{u}_{0}-{u}_{1}},\,{u}_{1}=0.05,$$

13 $$Z=j[X+M\,{\rm{Re}}({\psi }_{rad}(x,y)\,{\psi }_{ref}^{\ast }(x,y){e}^{j{k}_{0}(x\sin {\theta }_{0}\cos {\phi }_{0}+y\sin {\theta }_{0}\sin {\phi }_{0}})]$$

where *v*_0_ = 0.07, *u*_0_ = 0.42 and $({\theta }_{0},{\phi }_{0})=({20}^{\circ },{0}^{\circ })$. The desired object wave is depictedin Fig. 5a. Similar to the previous part, the amplitude and phase of the overall tangential electric field in the hologram plane as well as the corresponding impedance distribution is calculated and plotted in Fig. 5b up to 5d.

**Figure 6:**
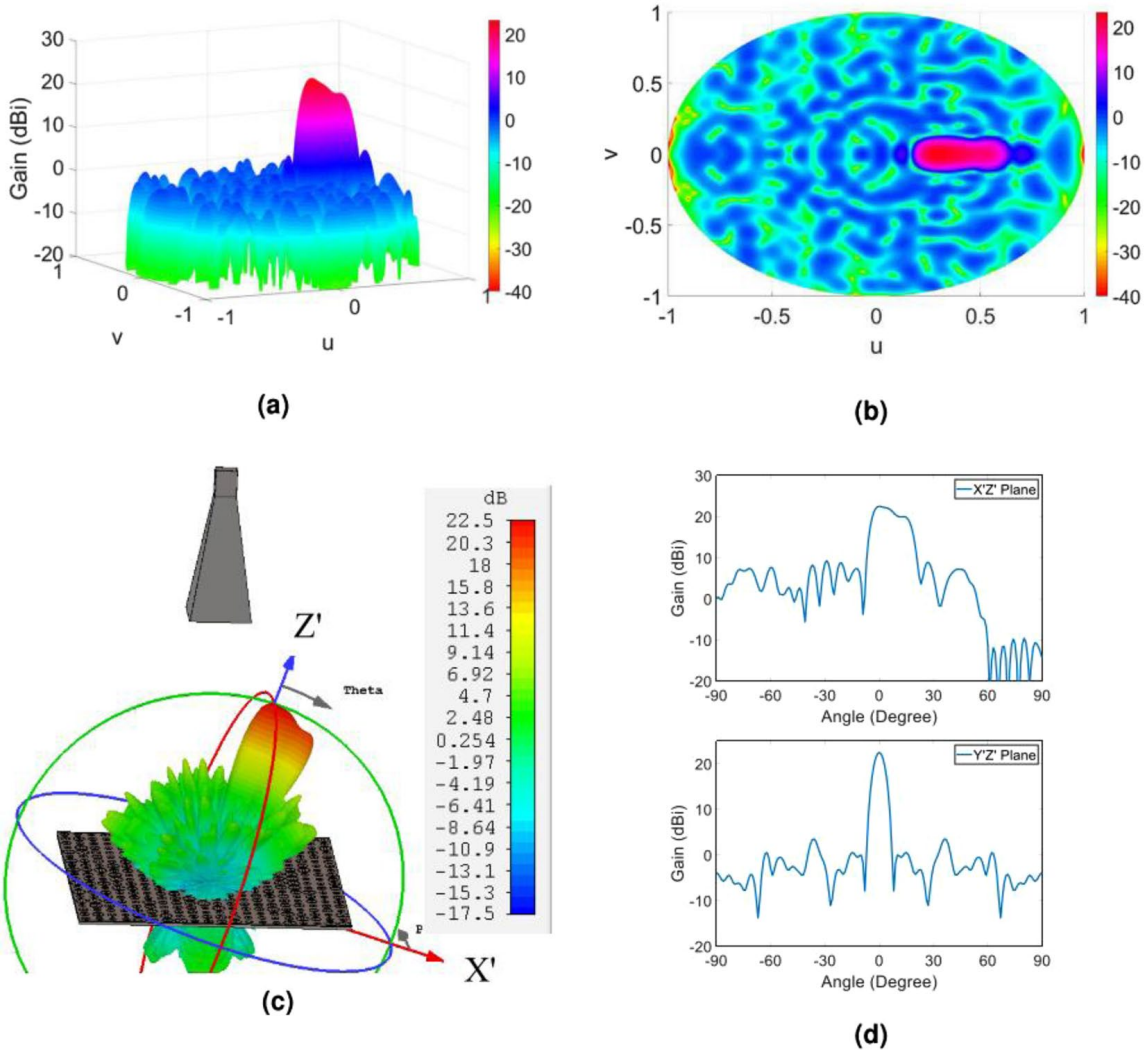
(**a**) Three-dimensional and (**b**) two-dimensional display of theoretical results of the object wave in the far field; (**c**) three-dimensional demonstration of the full wave simulation result of the object wave in CST software; (**d**) demonstration of the object wave in the two principal planes of the hologram. Note that the first plot of (**d**) is demonstrated in X′Z′-plane and the later plot is shown in the plane which is perpendicular to X′Z′-plane and contain the main beam direction that the peak gain occurs (i.e., *θ* = 20°, where *θ* is the polar angle in XYZ coordinate system).

Theoretical and simulated far-filed patterns for this case are shown in Fig. 6. It is clear that using the convolution theorem and applying an exponential function with uniform amplitude and linear phase variation to the hologram impedance profile lead to converting a non-tilted objective wave to a tilted object wave without doing any extra computational cost, which is usually observed in the classical synthesis method by virtue of aligning the main principal axis of the original problem to the beam direction and solving a more complicated problem.

#### Wide angle isoflux beam

To further verify the proposed method, we implement a well-known pattern named isoflux which is widely used in satellite systems. In the following, the corresponding object wave is introduced.

14 $${G}_{copol}^{far\,field(Y)}=[6\,{\bf{rect}}(u/{u}_{0},v/{v}_{0})-5]\times [\mathrm{log}(\zeta /{\rm{\max }}(\zeta ))+18]$$

15 $$\zeta =\sigma |\cos (\arcsin (v))|-v\sqrt{1-{\sigma }^{2}},\,\sigma =1+h/{R}_{e},\,{R}_{e}=6371\,km,\,h=2000\,km$$

**Figure 7:**
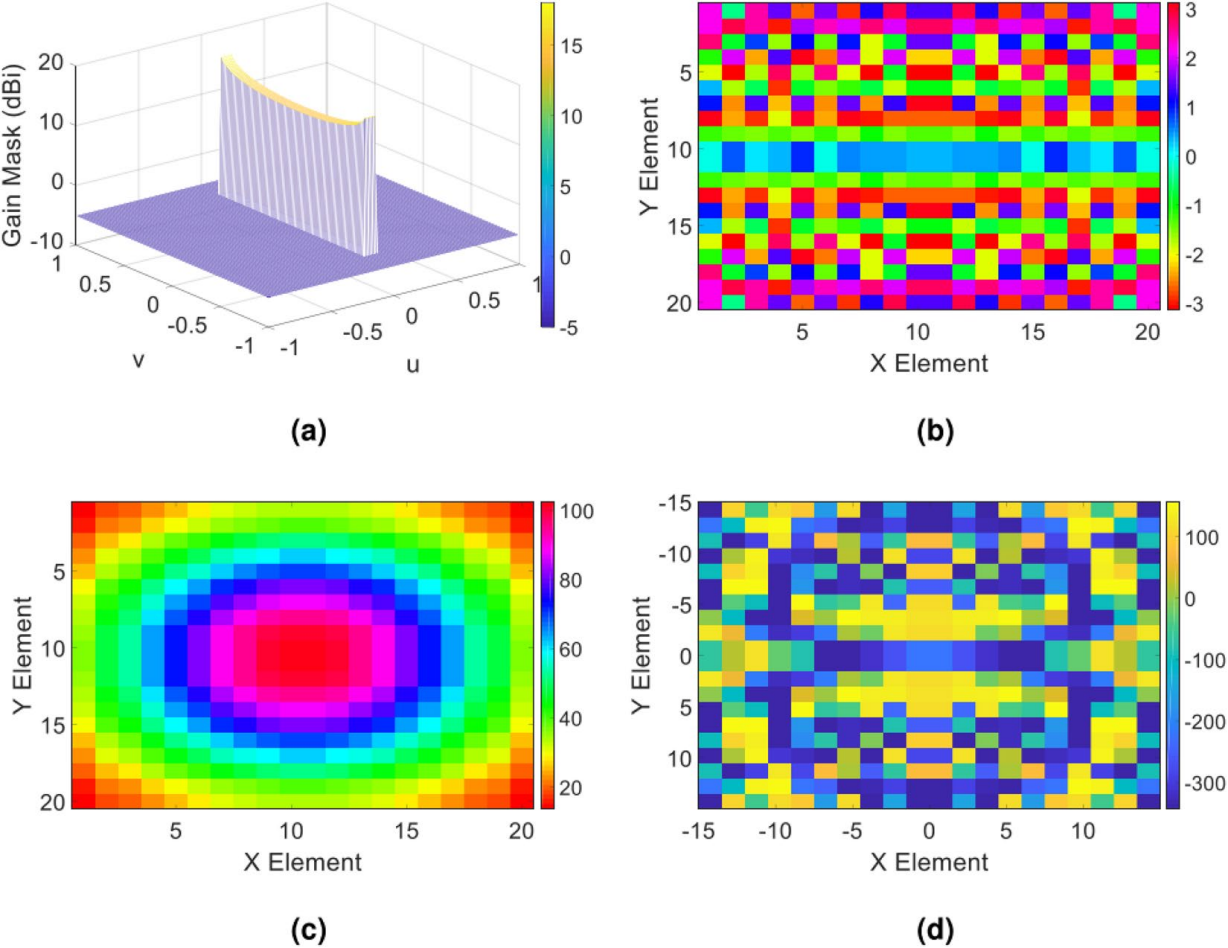
(**a**) The expected object wave in the (u, v) plane; (**b**) the phase and (**c**) amplitude of overall tangential electric field in the hologram plane; (**d**) the impedance distribution of the hologram obtained from the Eq. (1).

The co-pol gain for the type of the isoflux beam presented here is defined in accordance with the one expressed in recently reported research^45^. In (15), the parameters *R*_*e*_ and *h* are assigned to the earth radius and satellite altitude, respectively. We consider the boundary of the isoflux beam by defining *v*_0_ = 0.75 and *u*_0_ = 0.05, showing that the beam has a wide covered area around 97.2° in the u = 0 plane (or equivalently the yz-plane). The desired object wave is depicted in Fig. 7a. Again, the requirements for the hologram implementation are provided by following the extraction process of the impedance distribution outlined in the previous. The amplitude and phase of the overall tangential electric field in the hologram plane as well as the corresponding impedance distribution is plotted in Fig. 7b up to 7d.

**Figure 8:**
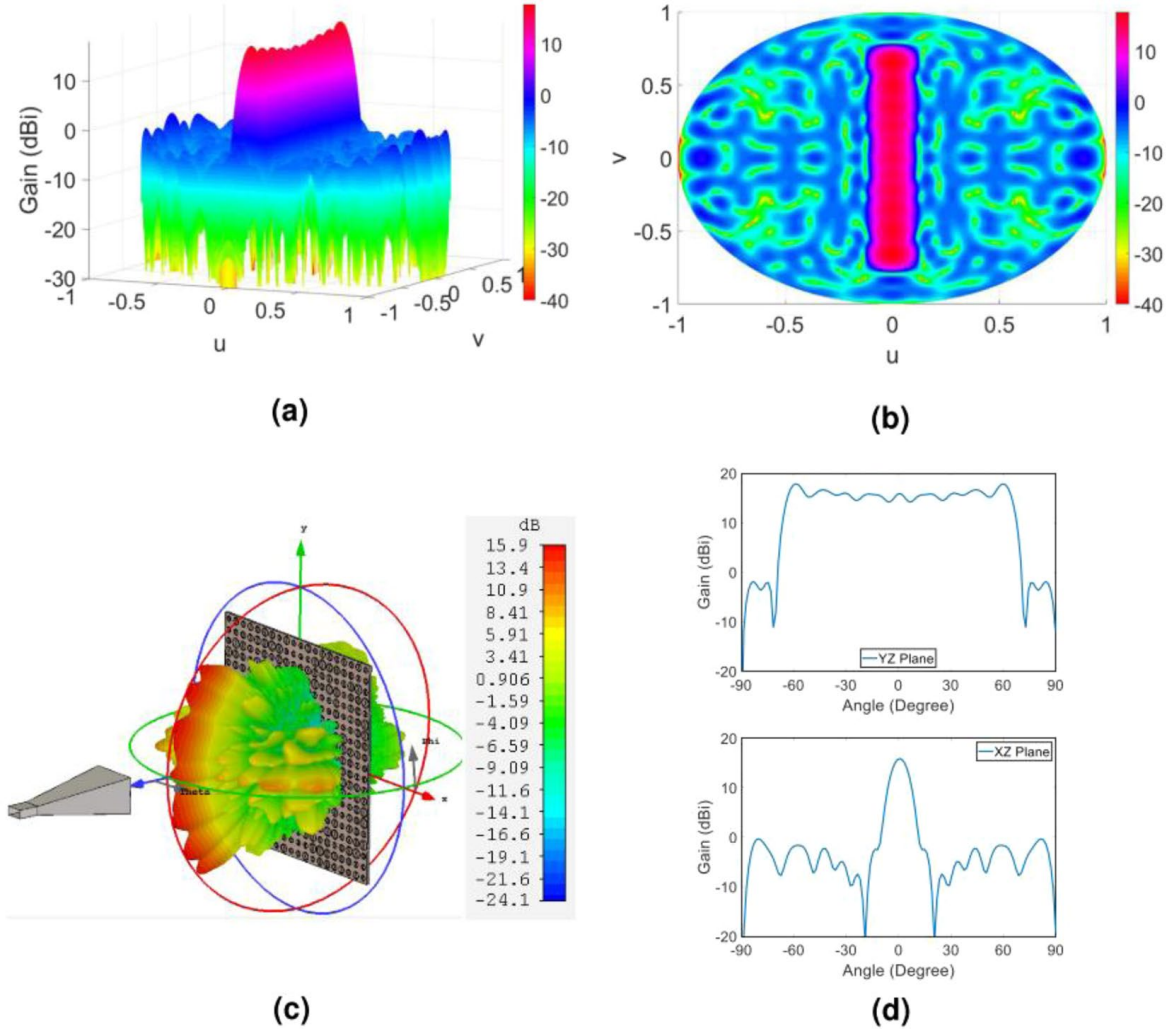
(**a**) Three-dimensional and (**b**) two-dimensional display of theoretical results of the object wave in the far field, (**c**) three-dimensional demonstration of the full wave simulation result of the object wave in CST software, (**d**) demonstration of the object wave in the two principal planes of the hologram. Note that the patterns demonstrated in (**d**) are related to the principal planes of YZ- and XZ-planes or u = 0 and v = 0, respectively.

Theoretical and simulation far-filed patterns for this case are shown in Fig. 8. We observe a significant agreement between the final radiated pattern and expected one.

#### Combination of two squared cosecant and pencil beam patterns

**Figure 9:**
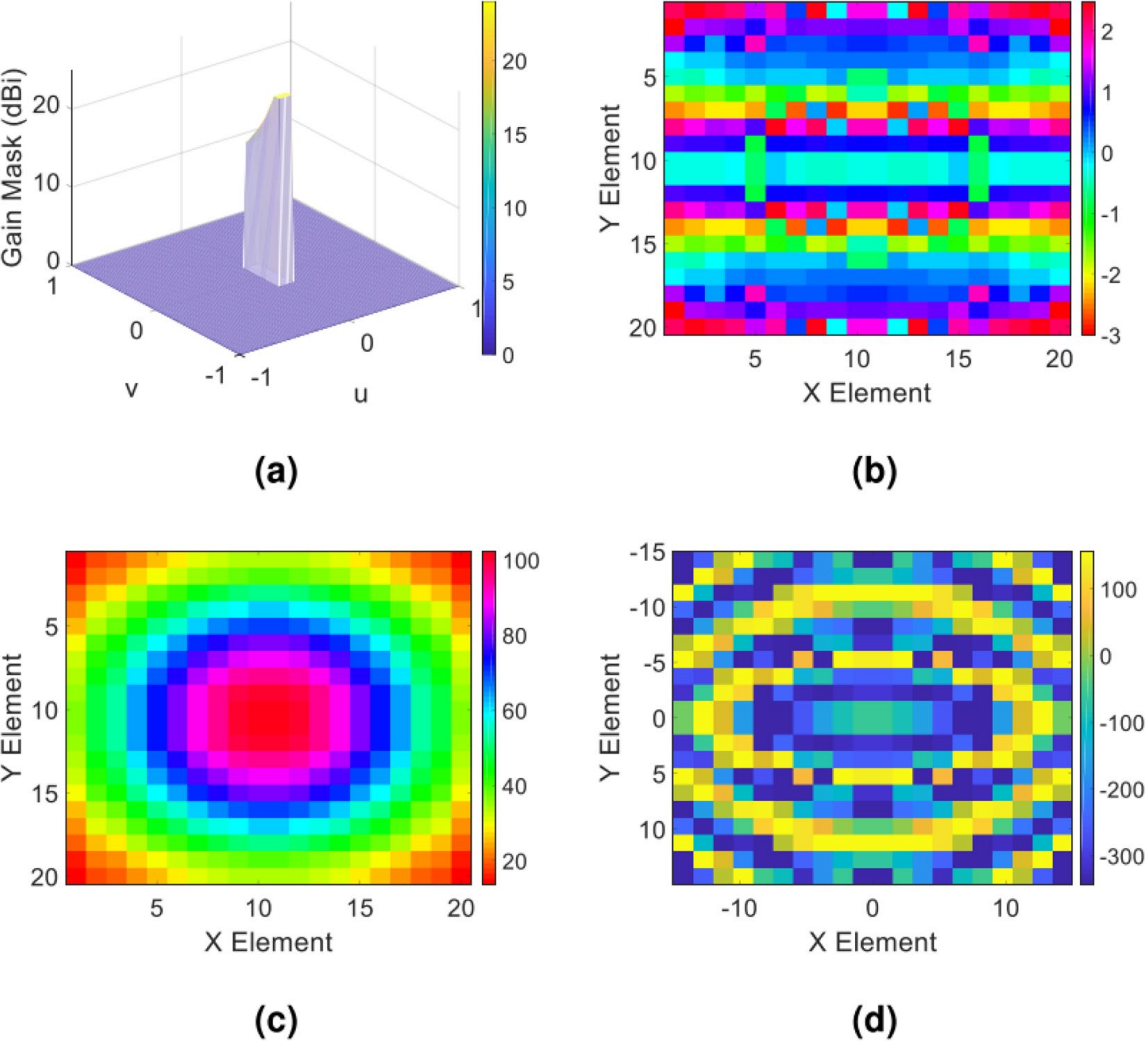
(**a**) The expected object wave in the (u, v) plane; (**b**) the phase and (**c**) amplitude of overall tangential electric field in the hologram plane; (**d**) the impedance distribution of the hologram obtained from the Eq. (1).

As a final example, we develop a more complicated pattern including two tilted squared cosecant beams and one ordinary pencil beam radiated in (*θ*_0_, *φ*_0_) = (0°, 0°). As before, it is enough to choose a non-tilted cosecant beam as the object wave and then use the superposition theory and convolution to specify the hologram impedance distribution. The non-tilted squared cosecant as the part of the desired object wave is depicted in Fig. 9a. In this regard, we can define

16 $${G}_{copol}^{far\,field(Y)}=[{\bf{rect}}(u/{u}_{0},v/{v}_{0})]\times $$

17 $$[10\,\mathrm{log}({|\csc (\arcsin (v^{\prime} ))|}^{2}/{\rm{\max }}({|\csc (\arcsin (v\text{'}))|}^{2}))+25]$$

18 $$v^{\prime} =u\times {\bf{rect}}\frac{v-{v}_{1}}{{v}_{0}-{v}_{1}},\,{v}_{1}=0.05\,,$$

And the impedance distribution is defined as follows

19 $$Z=j[X+\frac{M}{3}{\rm{Re}}({\psi }_{ref}^{\ast }[{\psi }_{rad}({e}^{j{k}_{0}(x\sin {\theta }_{1}\cos {\phi }_{1}+y\sin {\theta }_{1}\cos {\phi }_{1})}+{e}^{j{k}_{0}(x\sin {\theta }_{2}\cos {\phi }_{2}+y\sin {\theta }_{2}\cos {\phi }_{2})})+1])]$$

where *u*_0_ = 0.07, *v*_0_ = 0.42, ${\theta }_{1}={20}^{\circ },{\phi }_{1}={0}^{\circ }$, and ${\theta }_{2}={20}^{\circ },{\phi }_{2}={180}^{\circ }$. It is observed that the pencil beam direction is aligned with the z-axis. The amplitude and phase of the overall tangential electric field in the hologram plane as well as the corresponding impedance distribution is plotted in Fig. 9b up to 9d.

**Figure 10:**
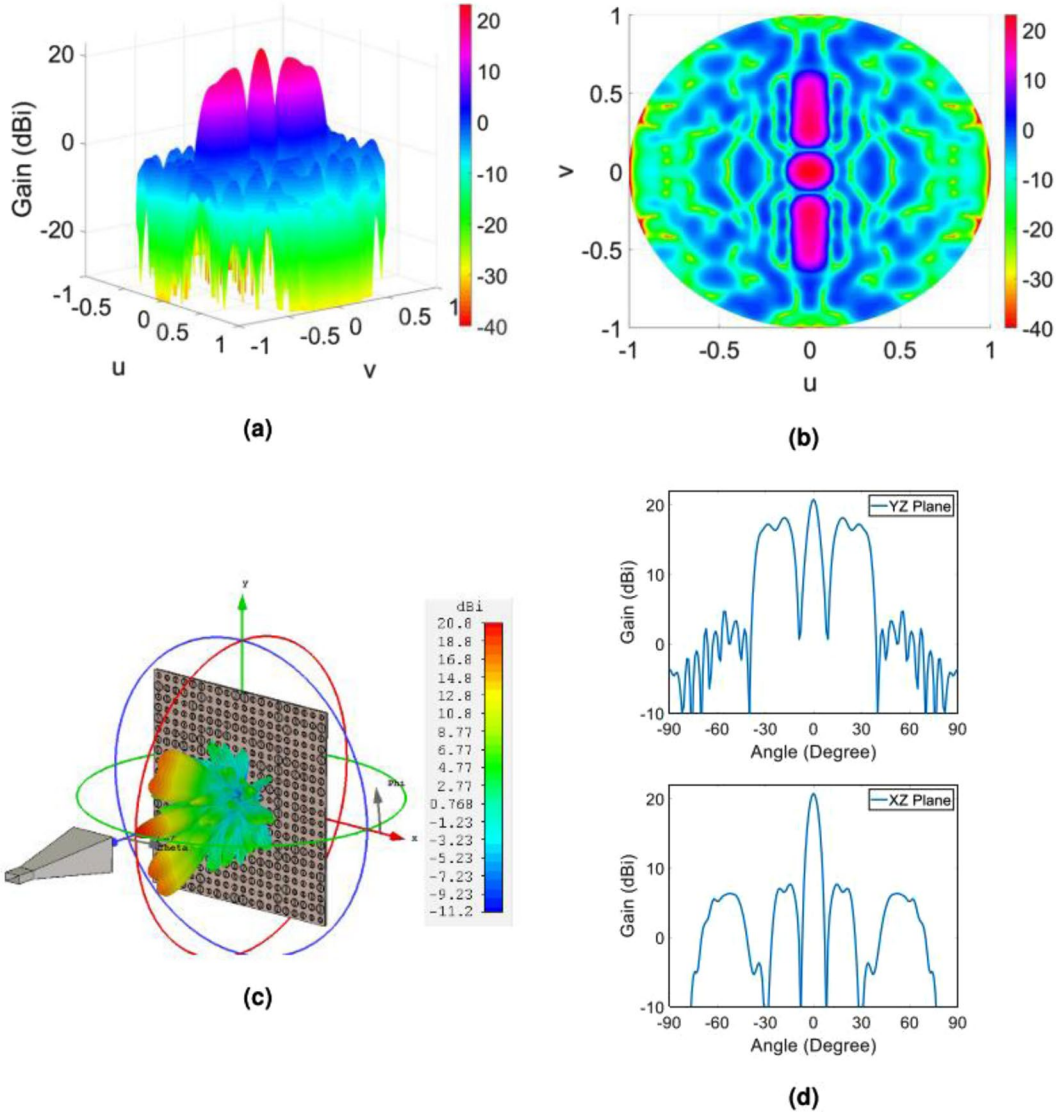
(**a**) Three-dimensional and (**b**) two-dimensional display of theoretical results of the object wave in the far field; (**c**) three-dimensional demonstration of the full wave simulation result of the object wave in CST software; (**d**) demonstration of the object wave in the two principal planes of the hologram. The principal planes in (**d**) are the YZ- and XZ-planes or u = 0 and v = 0, respectively.

Theoretical and simulation far-filed patterns for this case are shown in Fig. 10. It is evident that the overall shape of the far-field pattern (the reconstructed object wave) agrees with the desired waves defined as the object waves. Close examination shows that the main beam directions and peak gain of the reconstructed object waves are in accordance with the theoretical results.

### Fabrication and measurement

**Figure 11:**
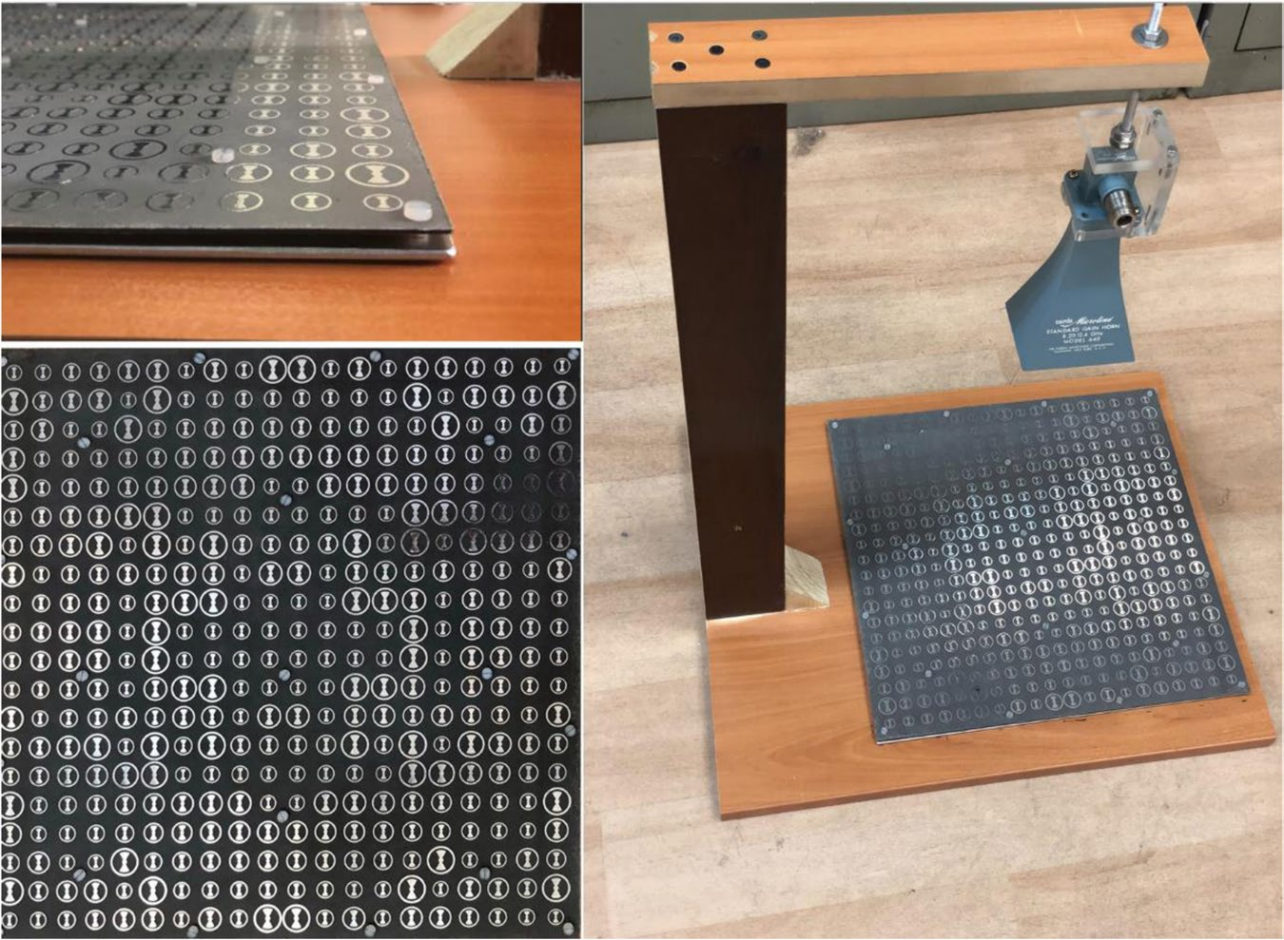
The fabricated three-beam hologram including two squared cosecant beams and one pencil beam.

**Figure 12:**
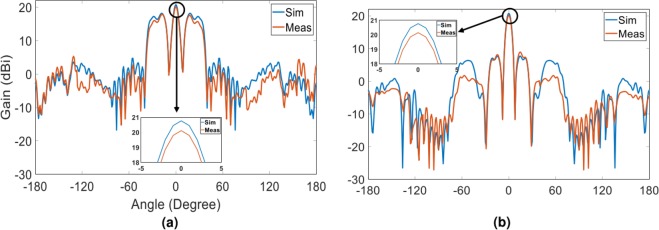
The comparison between the simulation and measurement results of the fabricated hologram in (**a**) phi = 90 and (**b**) phi = 0.

In order to experimentally validate the accuracy of the proposed method, the hologram designed and implemented in the last example was fabricated and the far-field radiation patterns were measured in the two principal planes and then compared with simulation results. Again, the hologram is composed of 400 unit cells with the capability of producing two tilted beams in the form of squared cosecant and one broadside pencil beam. Figure 11 demonstrates the fabricated hologram along with the measurement setup and reference wave emitter. Some plastic screw was employed to fix the applied 3 mm long spacers embedded between ground and dielectric layers. The experimental results were compared with simulation ones in the both principal planes as shown in Fig. 12. As can be observed in Fig. 12, there is a good agreement between the simulation and measurement results, specifically in the shaped region, verifying the proposed design method. The peak gain of simulation and measurement results are 20.77 dBi and 20 dBi.

The antenna efficiency, including the aperture efficiency and element loss, can be calculated from the far-field radiation pattern of antenna^43^. In summary, the following relation is employed to determine the antenna efficiency.

20 $$e=\frac{G(\theta ,\varphi )}{D(\theta ,\varphi )}=\frac{{P}_{rad}}{{P}_{feedHorn}}=\frac{{\int }_{0}^{2\pi }\,{\int }_{0}^{\pi }\frac{1}{2\eta }{|{E}_{rad}(\theta ,\varphi )|}^{2}\,\sin \,\theta \,d\theta \,d\varphi }{\frac{\pi {|{A}_{0}|}^{2}}{\eta }\frac{2}{2q+1}}$$

In (20), *G*(*θ*, *ϕ*) and *D*(*θ*, *ϕ*) are the gain and directivity of the antenna, respectively. The quantity *E*_*rad*_(*θ*, *ϕ*) is the radiated electric field in far zone, which is determined with CST MWS for all spherical angles of (*θ*, *ϕ*). In denominator of relation (20), *η* is the intrinsic impedance of the free space, q and *A*_0_ describe the analytical model of the pattern of the feed as (*A*_0_ cos^*q*^*θ*), which can be computed with CST MWS by comparing that analytical model with the radiated far-field pattern of the feed. In light of the above discussion, the antenna efficiency described in the last example will be obtained 55.89 %.

## Conclusion

A systematic method is presented to design a shaped-beam emitter reflector-based metasurface. The method combines the holography concept and convolution theorem together and introduces a novel synthesis method for shaping EM waves with flexible and continuous control of the beam direction without using any optimization algorithm and extra computational cost. Using the holography and convolution theorem gives the designer a new insight for implementing versatile radiation patterns from a new point of view. It is shown that the method is independent of the object shape and also the object number. Furthermore, the method has the potential to be used in such structures with simultaneous demonstration of several functionalities for the beam direction and beam shaping. This may be done by incorporating active devices as the realization approach of the hologram impedance profile.
